# Severity prediction for *Clostridium difficile* infection with ATLAS score – new data from a less developed country (Romania)

**DOI:** 10.1186/1471-2334-13-S1-O21

**Published:** 2013-12-16

**Authors:** Irina Crîşmaru, Șerban Benea, Gabriel Adrian Popescu

**Affiliations:** 1 Carol Davila University of Medicine and Pharmacy, Bucharest, Romania; 2 National Institute for Infectious Diseases “Prof. Dr. Matei Balş”, Bucharest, Romania

## Background

The emergence of ribotype 027 increased the severity of *Clostridium difficile* infection (CDI). Fast severity assessing plays an essential role in decreasing the mortality due to CDI; early surgery is the best intervention to decrease the lethality in severe CDI with toxic megacolon. Several severity scores were defined, including the ATLAS score, but the validation studies were performed in developed countries with structured medical practice. We evaluated the usefulness of ATLAS score and of its components as indicators for CDI severity (deaths, length-of-stay, costs).

## Methods

We calculated in a retrospective manner the ATLAS score for 170 consecutive patients with CDI, admitted in the National Institute for Infectious Diseases “Prof. Dr. Matei Balş” during a seven months period, September 2012 to March 2013. The mean and median values for parameters of ATLAS score as the score itself were compared for survivor (S; n=159 patients) and non-survivor (NS; n=11) groups.

## Results

The median value of ATLAS score was significantly higher in the non-survivors as in survivors: 6 versus 3, p=0.00002. The highest relative risk of death was associated with a value of ATLAS score of 5 or more, RR = 16.46 (95% CI: 3.69-73.46; p=0.0002). The patients in NS group were older: 79 years versus 64.1 years (p=0.000076), had a greater value of highest temperature: 38.7°C versus 37.9°C (p=0.00028), and a lower level of initial albuminemia: 2.33 g/dL versus 3.12 g/dL (p=0.00044). For the initial WBC count the value is greater in NS group, with almost defined statistical significance: 21,918/cmm versus 12,130/cmm (p=0.071). Concomitant systemic antibiotic treatment was prescribed in 5 of 11 patients in NS group and in 48 of 159 patients in S group, p=0.29; the overall concomitant systemic antibiotic use was 32.1%, greater than in the validation study of ATLAS score (19%). The mean duration of hospitalization in all patients was 12.5 days; the value of ATLAS score was also positively correlated to the length-of-stay, R^2^=0.922. The mean cost of medication was 2697.4±4685.55 RON (range 25-31254 RON); the value of ATLAS score had a good correlation with medication costs: R²=0.732, p=0.003.

## Conclusion

The ATLAS score confirmed its value as predictor of outcome in CDI patients, for lethality, lenght-of-stay and direct costs of medication. Among ATLAS score parameters, only simultaneous CDI treatment and systemic antibiotics use was not significantly different in S and NS groups, due to the large extent of systemic antibiotic treatment in our patients.

